# Anthropometry, sex, and age at diagnosis affect pulmonary blood volume quantification from computed tomography pulmonary angiography in pulmonary hypertension assessment

**DOI:** 10.3389/fcvm.2026.1815977

**Published:** 2026-05-11

**Authors:** Hakim Ghani, Muhunthan Thillai, Simon Walsh, Elliott Bussell, Martin Graves, Joanna Pepke-Zaba

**Affiliations:** 1 National Pulmonary Hypertension Centre, Pulmonary Vascular Disease Unit, Royal Papworth Hospital, Cambridge, United Kingdom; 2 University of Cambridge, Cambridge, United Kingdom; 3 Interstitial Lung Diseases Unit, Royal Papworth Hospital, Cambridge, United Kingdom; 4 Qureight Ltd., Cambridge, United Kingdom; 5 National Heart and Lung Institute, Imperial College, London, United Kingdom

**Keywords:** age, AI, anthropometry, CTPA, pulmonary blood volume, sex

## Abstract

**Introduction:**

The influence of anthropometrics, sex, and age at diagnosis on artificial intelligence (AI)–derived pulmonary blood volumes (PBV) from computed tomography pulmonary angiography (CTPA) remains poorly characterized. These physiological and biological determinants may affect PBV-based pulmonary hypertension (PH) prediction models.

**Methods:**

An AI-based segmentation model quantified pulmonary artery and vein volumes in a secondary CTPA analysis from the Cambridge PH Registry. PBV were modelled as functions of anthropometrics, sex, and age at diagnosis, adjusting for pulmonary vascular resistance (PVR), PH diagnostic category (group 1, 2, 3 or 4 PH, or no PH), and number of cardiac comorbidities. The impact of PBV normalization strategies, including anthropometrics, on sex-related differences was assessed. Multivariable linear regression evaluated associations between PBV and invasively measured PVR and cardiac output (CO), and the incremental predictive value of anthropometrics.

**Results:**

376 patients (median age 60 years; 57% female) investigated with right heart catheter were included: 120 pulmonary arterial hypertension, 30 group 2 PH, 79 group 3 PH, 102 chronic thromboembolic PH, and 45 without PH. Pulmonary artery volume increased with height, weight, body mass index (BMI), and body surface area (BSA) (all *p* < 0.001), with a stronger height-related effect in males. Older diagnosis age associated with larger pulmonary artery volume, particularly those with higher weight, BMI, and BSA (all *p* < 0.001). Pulmonary vein volume also increased with anthropometrics, but with older age disproportionately increasing volumes in females with higher weight, BMI, and BSA. PBV normalization to anthropometrics (height, weight, and BSA) attenuated sex-related differences (*p* < 0.001). In hemodynamic models, larger pulmonary artery volume and lower pulmonary vein volume were independently associated with higher PVR, while larger pulmonary vein volume was associated with higher CO (all *p* < 0.001). Inclusion of anthropometrics, particularly BSA, significantly improved prediction of PVR (*F* = 12.8, *p* < 0.001) and CO (F = 23.8, *p* < 0.001).

**Conclusion:**

AI-derived PBV from CTPA are shaped by complex interactions between anthropometrics, sex, and diagnosis age, with distinct effects on pulmonary arterial and venous compartments. Accounting for these determinants is essential for translating AI-quantified PBV into clinically intuitive PH prediction or phenotyping models.

## 1 Introduction

Pulmonary hypertension (PH) arises from a spectrum of etiologies which result in significant morbidity and mortality (1). Right heart catheterization (RHC) remains the gold standard for PH diagnosis by providing invasive cardiopulmonary hemodynamic measurements. However, the clinical evaluation of PH is inherently multimodal, where computed tomography (CT) has become an essential and widely accessible non-invasive component of the diagnostic and assessment pathway.

In routine clinical practice, CT assessment in PH is largely limited to a relatively small number of qualitative or semi-quantitative features (2, 3). This includes measurement of the main pulmonary artery diameter or pulmonary artery–to–aorta diameter ratio to estimate PH probability; visual assessment of cardiac chamber sizes to infer right- and/or left-sided dilatation; identification of organized thromboembolic disease as a PH etiology; and evaluation of pulmonary parenchyma for relevant lung diseases. Consequently, much of the quantitative vascular information contained within CT images is currently not routinely exploited.

Advances in artificial intelligence (AI) now enable automated segmentation of the pulmonary vasculature from CT images, allowing quantitative estimation of pulmonary blood volumes (PBV) (4–9). Such measurements have shown to offer additional pathophysiological insight from CT features in PH which are currently not captured during standard clinical assessment. This holds potential value for leveraging CT in PH screening, risk stratification, phenotyping, and assessment of treatment response.

The successful clinical translation of AI-based CT metrics depends on their transparent application and reproducibility, which is underpinned by high-quality, diverse, and representative training data to promote equitable clinical utility (10–13). Therefore, embedding AI-derived PBV metrics into explainable PH predictive models require an understanding of how physiological factors modulate vascular volumes (14–16). In particular, the effects of anthropometric characteristics [height, weight, body mass index (BMI), and body surface area (BSA)], biological sex, and age at diagnosis on CT-derived PBV remain poorly characterized. This knowledge gap is likely driven by the limited size and heterogeneity of previously studied PH cohorts, restricting detailed interaction analyses. Without appropriate contextualization or normalization, such factors may confound biases in PBV-based PH predictive models and limit clinical interpretability (10–13).

In this study, we investigated the influence of anthropometrics, sex, and diagnosis age on AI-quantified pulmonary artery and vein volumes derived from CT pulmonary angiography (CTPA) in a large cohort of well-characterized patients assessed for PH at a UK national PH referral center. We additionally assessed PBV normalization strategies to address expected sex-related differences in pulmonary vascular volume. Furthermore, we evaluated whether CT-derived PBV as predictors of invasively measured pulmonary vascular resistance (PVR) and cardiac output (CO) were influenced by anthropometrics. These analyses aim to support the translation of AI-quantified CTPA-derived PBV into robust, clinically intuitive, and physiologically grounded biomarkers for PH prediction and phenotyping.

## 2 Methods

### 2.1 Patient cohort and clinical data

**Figure 1:**
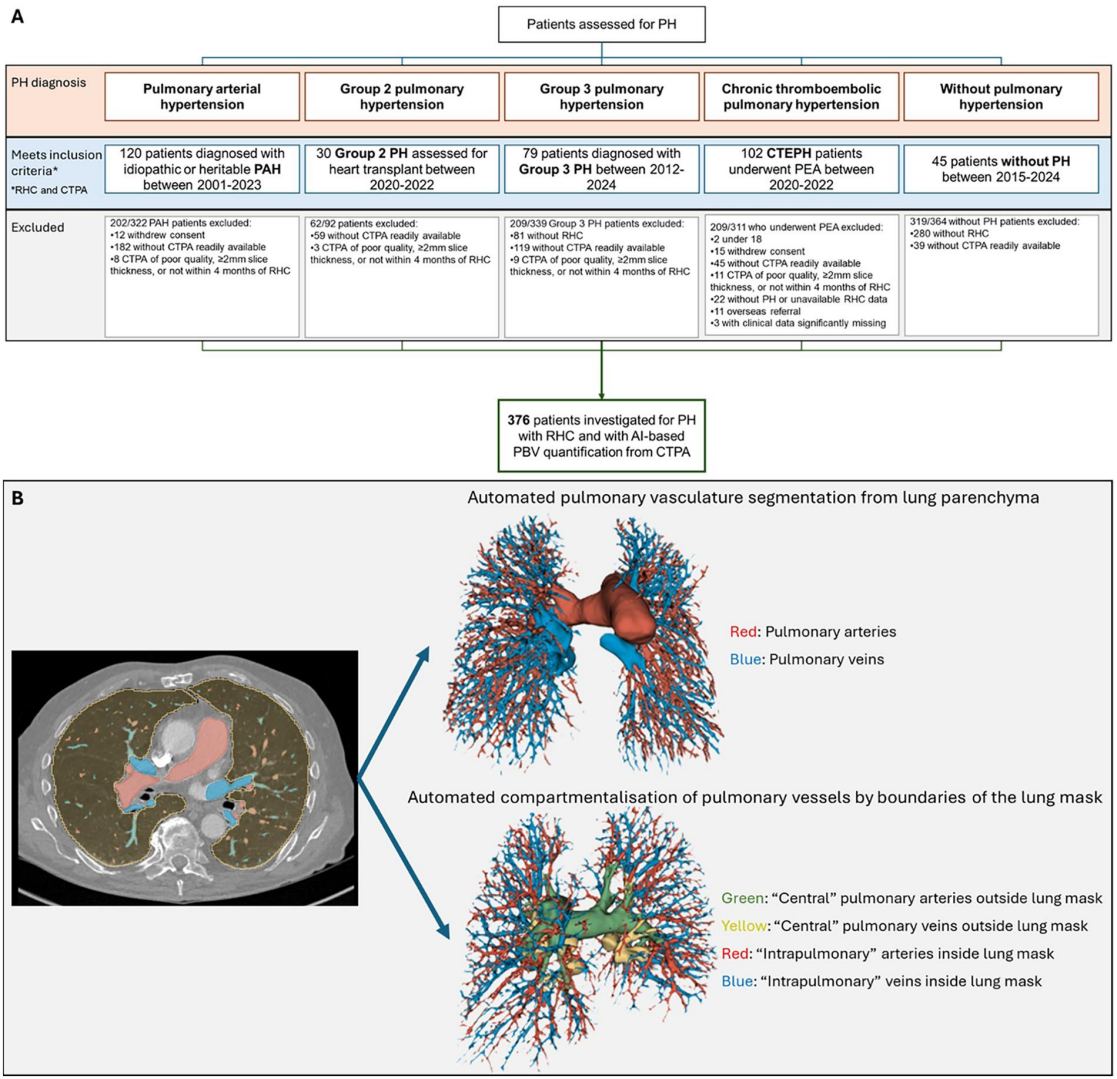
**(A)** Flow chart of patients recruited with inclusion and exclusion criteria. **(B)** Automated pulmonary vasculature segmentation from lung parenchyma and compartmentalization by boundaries of the lung mask from CTPA images. CTEPH, chronic thromboembolic pulmonary hypertension; CTPA, computed tomography pulmonary angiography; PAH, pulmonary arterial hypertension; PH, pulmonary hypertension; RHC, right heart catheter.

Patients who were investigated for suspected PH with RHC and had CTPA available at Royal Papworth Hospital were included. Consecutive pulmonary arterial hypertension (PAH), group 2 PH, group 3 PH, chronic thromboembolic PH (CTEPH), and without PH patients were identified by screening the prospective CAPHTURE (Cambridge PH Registry) database. PH was defined as mean pulmonary arterial pressure (mPAP) of >20 mmHg on RHC. The study was approved by the National Health Service Health Research Authority (REC reference: 23/HRA/3260). An extended description of recruitment is available in the supplement (Extended methods). The study protocol was registered with the Open Science Framework (OSF; registration DOI: 10.17605/OSF.IO/RPVZH). Figure 1A illustrates patient recruitment with inclusion and exclusion criteria.

### 2.2 Study objectives

In patients investigated for PH, this study aimed to use AI-based CTPA-derived PBV quantification to:

1. Characterize the independent and interacting effects of anthropometrics, biological sex, and age at PH diagnosis on pulmonary artery and vein volumes.
2. Evaluate whether sex-related differences in PBV are attenuated by normalization to anthropometrics, total pulmonary vessel volume, or lung volume.
3. Determine whether anthropometric factors influence the associations between PBV and invasively measured PVR and CO

### 2.3 CTPA acquisition, automated segmentation, and pulmonary blood volume quantification

CTPA images from multiple secondary and tertiary hospitals were anonymized in DICOM format. As CTPA images from multiple hospitals were analyzed as part of an observational study, acquisition protocols were not standardized.

A previously published AI-based tool (Vascul8™, Qureight Ltd, Cambridge, UK) was used for pulmonary vasculature segmentation from CTPA images (4). Automated pulmonary vasculature segmentation from lung parenchyma with differentiation of arteries and veins were performed by a three-dimensional convolutional neural network-based intensity thresholding (Figure 1B). Pulmonary vessels were segmented from the cardiac border up to the last visible distal generation on CTPA. We had previously reported the performance of automated pulmonary vascular segmentations (Vascul8) by the amount of overlap/similarity with manual segmentations of CTPA images in 142 patients, achieving a Dice score of 0.77 (4). Pulmonary artery, pulmonary vein and total vessel blood volume measurements were compared between the automated and manual segmentations, each resulting in root mean square error percentages of <10%. As previously published AI-based vascular segmentation tool was utilized, re-assessment of performance was not performed for this study. Extended descriptions of CTPA processing and Vascul8™ are in the supplement.

Outputs of automated segmentations from CTPA were reviewed for quality assurance by radiologists. Pulmonary arteries and veins were additionally automatically compartmentalized as previously published by anatomical boundaries of the lung mask (4). These are labelled as “central” (up to proximal lobar vessels) and “intrapulmonary” (predominantly segmental and subsegmental), and blood volumes evaluated separately (Figure 1B). A total of 12 CTPA-based metrics were measured through Vascul8^TM^ and blinded to all other data collected in this study (online supplement).

### 2.4 Statistical analysis

Statistical analyses were performed using R (version 4.3.3). Continuous variables were compared between groups using non-parametric Wilcoxon rank-sum tests, and categorical variables using Fisher's exact test. Associations between anthropometric and CTPA-derived measures were assessed using Spearman correlation coefficients (rₛ), with adjustment for sex, age at diagnosis, PVR, and PH diagnostic category (group 1, 2, 3 or 4 PH, or without PH). False discovery rate (FDR) correction was applied, with adjusted *p*-values < 0.05 considered statistically significant.

PBV were modelled using multivariable linear regression as functions of anthropometrics (height, weight, BMI, or BSA), sex, and age at diagnosis, including two- and three-way interaction terms. Models were adjusted for PVR, PH diagnostic category, and number of cardiac comorbidities. Sex-related differences between raw and normalized PBV were estimated from multivariable linear regression. Non-parametric bootstrapping with resampling was performed to quantify the attenuation of sex-related effects after PBV normalization in standardized (z-score) units.

Separate multivariable linear regression models were used to evaluate associations between standardized pulmonary artery and vein volumes and invasively measured PVR and CO, adjusting for age at diagnosis and sex. Nested models including anthropometric measures were compared using analysis of variance (ANOVA) to assess incremental predictive value and improvement in model fit. Further details of the statistical analysis are provided in the supplement.

## 3 Results

### 3.1 Patient cohort and correlations

A total of 376 patients investigated for PH (median age 60 years, 57% female) with RHC were included: 120 pulmonary arterial hypertension, 30 group 2 PH, 79 group 3 PH, 102 CTEPH, and 45 without PH. Table 1 illustrates baseline clinical characteristics.

**Table 1:** Baseline characteristics.

	All, *n* = 376	Female	Male
Pulmonary hypertension			
*n*	331	178	153
Age,years	59 [46, 70]	56 [43, 70]	63 [51, 71]
Height, cm	167 [160, 174]	161 [157, 165]	173 [169, 179]
Weight, kg	79 [68, 92]	74 [63, 86]	85 [75, 97]
BMI, kg/m^2^	28 [24, 33]	29 [24, 34]	28 [24, 32]
BSA, m^2^	1.92 [1.75, 2.09]	1.82 [1.67, 1.98]	2.03 [1.88, 2.17]
mPAP, mmHg	46 [38, 54]	48 [38, 56]	46 [38, 52]
CO, L/min	3.9 [3.1, 4.8]	3.7 [2.9, 4.5]	4.1 [3.4, 5]
CI, L/min/m^2^	2.1 [1.7, 2.5]	2.1 [1.7, 2.5]	2.1 [1.7, 2.5]
PVR, dynes.s^−1^.cm^−1^	693 [475, 998]	816 [520, 1,108]	584 [427, 854]
PCWP, mmHg	10 [8, 13]	10 [7, 13]	11 [8, 12]
6MWD, m	292 [182, 360]	292 [182, 360]	336 [232, 405]
WHO FC, *n*			
I	1 (0.3)	0	1 (0.6)
II	36 (10.9)	17 (9.5)	19 (12.4)
III	268 (80.9)	144 (81)	124 (81)
IV	26 (7.9)	17 (9.5)	9 (6)
NTproBNP, pg/mL	1,154 [293, 2,842]	1,203 [312, 2,558]	1,003 [279, 3,295]
FEV1, %	81 [67, 93]	80 [69, 95]	83 [64, 92]
FVC, %	91 [80, 105]	92 [80, 106]	91 [80, 102]
DLCO, %	60 [38, 73]	61 [44, 72]	59 [32, 76]
KCO, %	72 [52, 87]	72 [59, 87]	72 [45, 87]
PH diagnoses, *n*			
PAH	120 (36)	80 (45)	40 (26)
Group 2 PH	30 (9)	13 (7)	17 (11)
Group 3 PH	79 (24)	36 (20)	43 (28)
CTEPH	102 (31)	49 (28)	53 (35)
No pulmonary hypertension			
*n*	45	38	7
Age,years	67 [54, 75]	69 [55, 76]	56 [48, 69]
Height, cm	164 [158, 171]	163 [158, 168]	175 [174, 185]
Weight, kg	73 [65, 86]	70 [64, 80]	85 [75, 91]
BMI, kg/m^2^	27 [24, 30]	28 [24, 31]	25 [25, 29]
BSA, m^2^	1.71 [1.8, 1.98]	1.78 [1.7, 1.91]	1.99 [1.89, 2.15]
mPAP, mmHg	18 [15, 20]	18 [15, 20]	18 [16, 20]
CO, L/min	5.1 [4, 6.2]	5.2 [4, 6.1]	4.6 [4.2, 7]
CI, L/min/m^2^	2.8 [2.3, 3.3]	2.8 [2.4, 3.3]	2.5 [2.3, 3.3]
PVR, dynes.s^−1^.cm^−1^	139 [107, 170]	138 [107, 172]	139 [106, 145]
PCWP, mmHg	8 [6, 10]	9 [6, 10]	8 [7, 10]
6MWD, m	340 [232, 410]	334 [224, 402]	395 [356, 585]
WHO FC, *n*			
I	7 (15.6)	3 (8)	4 (57)
II	19 (42.2)	16 (42)	3 (43)
III	19 (42.2)	19 (50)	0
IV	0	0	0
NTproBNP, pg/mL	187 [83, 354]	194 [88, 391]	152 [85, 240]
FEV1, %	91 [83,102]	92 [84, 104]	89 [84, 99]
FVC, %	103 [89, 120]	102 [88, 114]	119 [105, 126]
DLCO, %	77 [63, 87]	77 [62, 86]	77 [72, 102]
KCO, %	81 [65, 97]	82 [65, 98]	75 [71, 83]

Results in median [IQR] or percentage ().

BMI, body mass index; BSA, body surface area; CTEPH, chronic thromboembolic pulmonary hypertension; WHO FC, World Health Organization functional class; mPAP, mean pulmonary artery pressure; CO, cardiac output; CI, cardiac index; PAH, pulmonary arterial hypertension; PH, pulmonary hypertension; PVR, pulmonary vascular resistance; PCWP, pulmonary capillary wedge pressure; 6MWD, 6 min walk distance.

**Figure 2:**
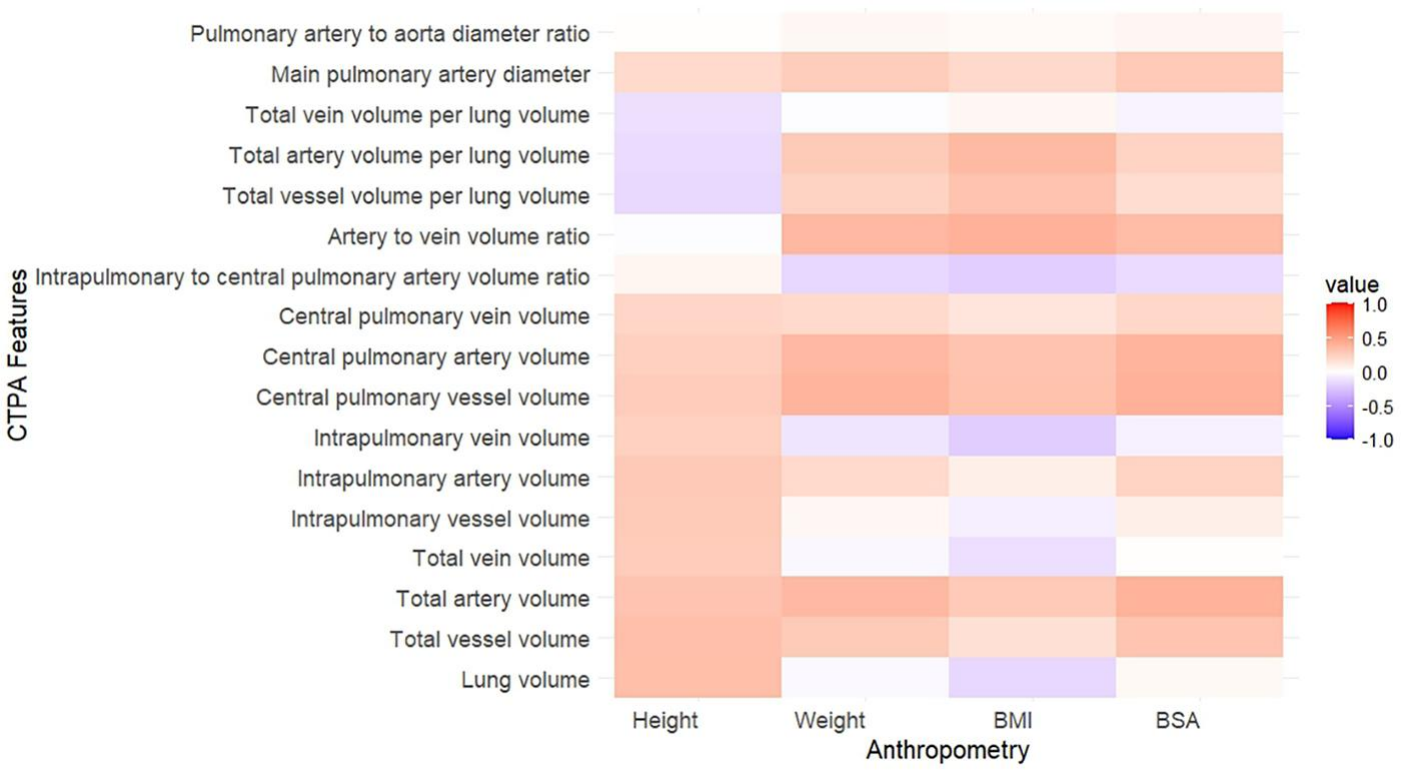
Heatmap illustrating the correlations between CTPA metrics and anthropometrics adjusted for biological sex, age at diagnosis, pulmonary vascular resistance, and PH diagnostic category. BMI, body mass index; BSA, body surface area.

After adjustment for sex, age at diagnosis, PVR, PH diagnostic category, the strongest correlations were seen between BSA and central pulmonary vessel volume (*r*_s_ = 0.4, *p* < 0.001), total artery volume (*r*_s_ = 0.39, *p* < 0.001), and central pulmonary artery volume (*r*_s_ = 0.38, *p* < 0.001) (Figure 2 and Supplementary Table S1). Pulmonary vein volume correlated strongest with height (*r*_s_ = 0.27, *p* < 0.001).

In multivariable models adjusted for PVR, PH diagnostic category, and cardiac comorbidity, biological sex and age at diagnosis were consistent independent predictors of pulmonary artery volume (both *p* < 0.001) and pulmonary vein volume (sex: *p* = 0.013 to <0.001; diagnosis age: *p* = 0.002–0.014) (Tables 2, 3). Male sex and older diagnosis age were associated with higher pulmonary artery and vein volumes. Anthropometric measures—including height (*p* < 0.001), weight (*p* < 0.001), and BSA (*p* < 0.001)—were each independently associated with pulmonary artery volume when modelled separately, where BMI (*p* = 0.004) demonstrated the weakest association (Table 2). Similarly, height (*p* < 0.001), weight (*p* < 0.001), and BSA (*p* < 0.001) were each independently associated with pulmonary vein volume when modelled separately, with BMI (*p* = 0.037) demonstrating the weakest association (Table 3).

**Table 2:** Multivariable linear regression models of pulmonary artery volume for each anthropometric measure separately, including sex and age at diagnosis.

Variables	Height model			Weight model			BMI model			BSA model		
	β ± SE	t	*p*	β ± SE	t	*p*	β ± SE	t	*p*	β ± SE	t	*p*
Sex (male)	21.7 ± 5	4.3	<0.001	42.1 ± 4	10.5	<0.001	49.0 ± 4	12	<0.001	36.4 ± 4	8.9	<0.001
Diagnosis age	0.75 ± 0.1	5.9	<0.001	0.71 ± 0.1	5.39	<0.001	0.59 ± 0.1	4.46	<0.001	0.75 ± 0.1	5.8	<0.001
Height	1.88 ± 0.2	7.6	<0.001	–	–	–	–	–	–	–	–	–
Weight	–	–	–	0.66 ± 0.1	5.6	<0.001	–	–	–	–	–	–
BMI	–	–	–	–	–	–	0.98 ± 0.3	2.94	0.004	–	–	–
BSA	–	–	–	–	–	–	–	–	–	61.46 ± 9	6.8	<0.001

Adjusted for pulmonary vascular resistance, pulmonary hypertension (PH) diagnostic category (group 1, 2, 3 or 4 PH, or without PH), and number of cardiac comorbidities.

BMI, body mass index; BSA, body surface area.

**Table 3:** Multivariable linear regression models of pulmonary vein volume for each anthropometric measure separately, including sex and age at diagnosis.

Variables	Height model			Weight model			BMI model			BSA model		
	β ± SE	t	*p*	β ± SE	t	*p*	β ± SE	t	*p*	β ± SE	t	*p*
Sex (male)	1.9 ± 0.7	2.49	0.013	3.4 ± 0.6	5.92	<0.001	4.1 ± 0.6	7.09	<0.001	3 ± 0.60	4.96	<0.001
Diagnosis age	0.05 ± 0.02	2.87	0.004	0.05 ± 0.02	2.76	0.006	0.05 ± 0.02	2.47	0.014	0.06 ± 0.02	3.11	0.002
Height	0.2 ± 0.04	4.02	<0.001	–	–	–	–	–	–	–	–	–
Weight	–	–	–	0.06 ± 0.02	3.38	<0.001	–	–	–	–	–	–
BMI	–	–	–	–	–	–	0.1 ± 0.05	2.1	0.037	–	–	–
BSA	–	–	–	–	–	–	–	–	–	5.3 ± 1.3	4.02	<0.001

Adjusted for pulmonary vascular resistance, pulmonary hypertension (PH) diagnostic category (group 1, 2, 3 or 4 PH, or without PH), and number of cardiac comorbidities.

BMI, Body mass index; BSA, Body surface area.

### 3.2 Height-sex-diagnosis age interaction

**Figure 3:**
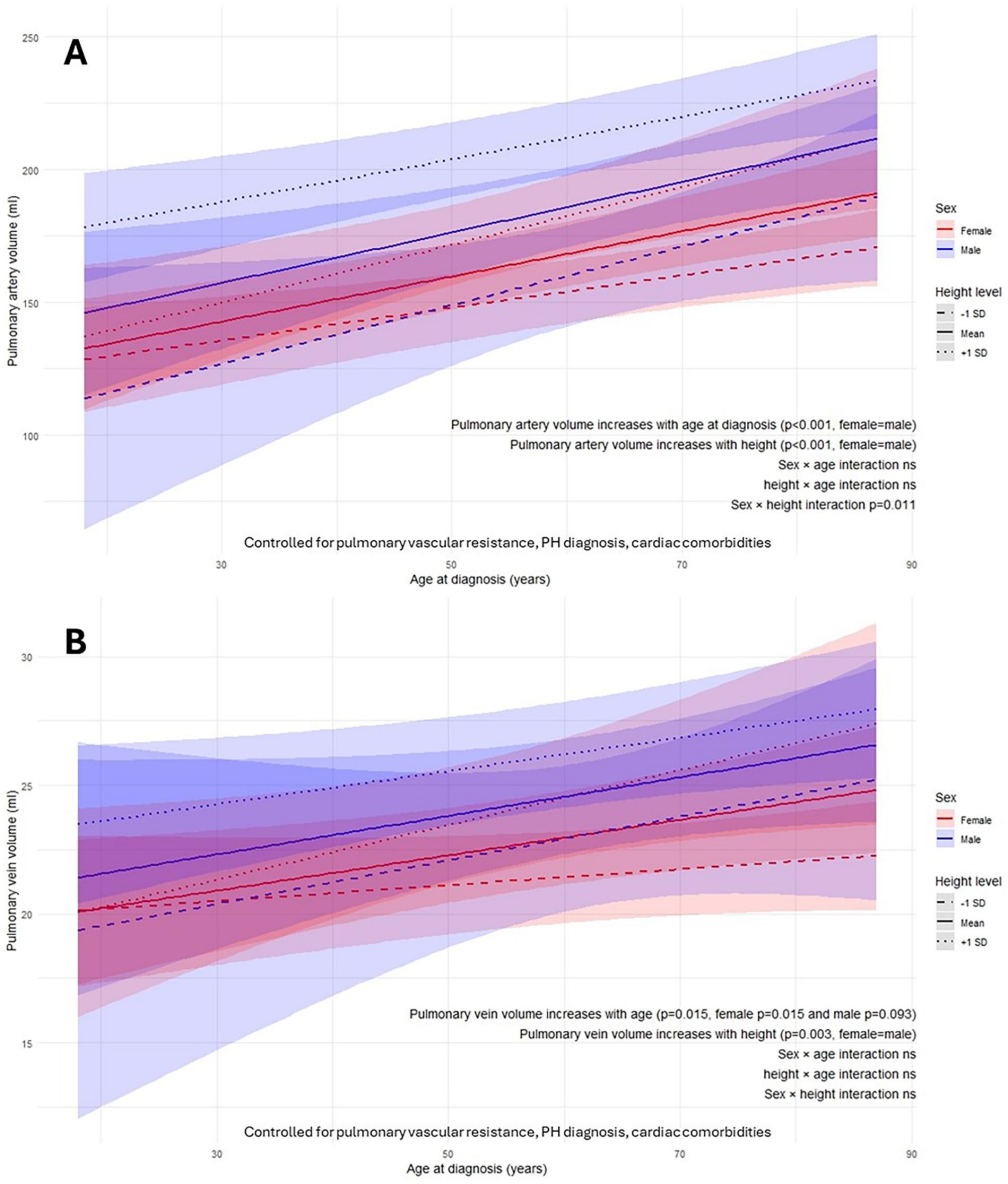
Height-sex-age at diagnosis interactions on AI-quantified pulmonary artery volume **(A)** and pulmonary vein volume **(B)** from computed tomography pulmonary angiography. ns, not significant; p, *p*-value; SD, standard deviation.

Pulmonary artery volume increased with height and age at diagnosis (both *p* < 0.001) in both sexes (Figure 3A). The association between height and pulmonary artery volume was significantly stronger in males than females (sex–height interaction *p* = 0.011).

Pulmonary vein volume increased with height in both sexes (*p* = 0.003) and with age at diagnosis in females (*p* = 0.015) (Figure 3B). Although males showed a similar age-related trend, this did not reach statistical significance (*p* = 0.093). Sex did not significantly modify the effects of height or diagnosis age on pulmonary vein volume (sex-height and sex-age interactions *p* = 0.792 and *p* = 0.905 respectively).

### 3.3 Weight-sex-diagnosis age interaction

**Figure 4:**
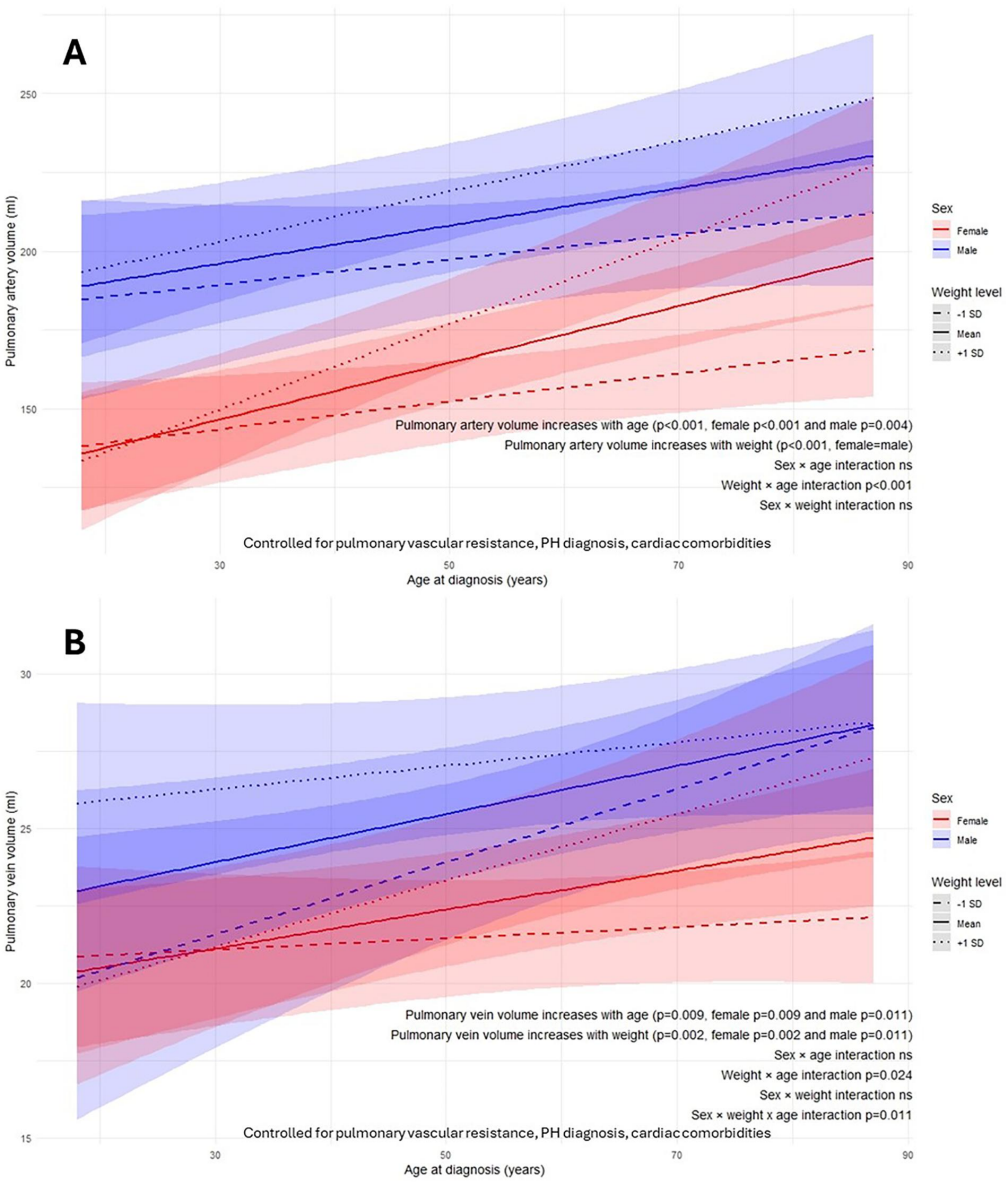
Weight-sex-age at diagnosis interactions on AI-quantified pulmonary artery volume **(A)** and pulmonary vein volume **(B)** from computed tomography pulmonary angiography. Ns, not significant; p, *p*-value; SD, standard deviation.

Pulmonary artery volume increased overall with weight and age at diagnosis (both *p* < 0.001) in both sexes (Figure 4A). There was significant diagnosis age–weight interaction indicating that the association between weight and pulmonary artery volume was stronger in patients at older age at diagnosis (age-weight interaction *p* < 0.001).

Pulmonary vein volume increased overall with weight (*p* = 0.002) and with age at diagnosis (*p* = 0.009) in both sexes (Figure 4B). The weight-related increase in pulmonary vein volume at older diagnosis ages was significantly greater in females than males (sex–weight–age interaction *p* = 0.011).

### 3.4 BMI-sex-diagnosis age interaction

**Figure 5:**
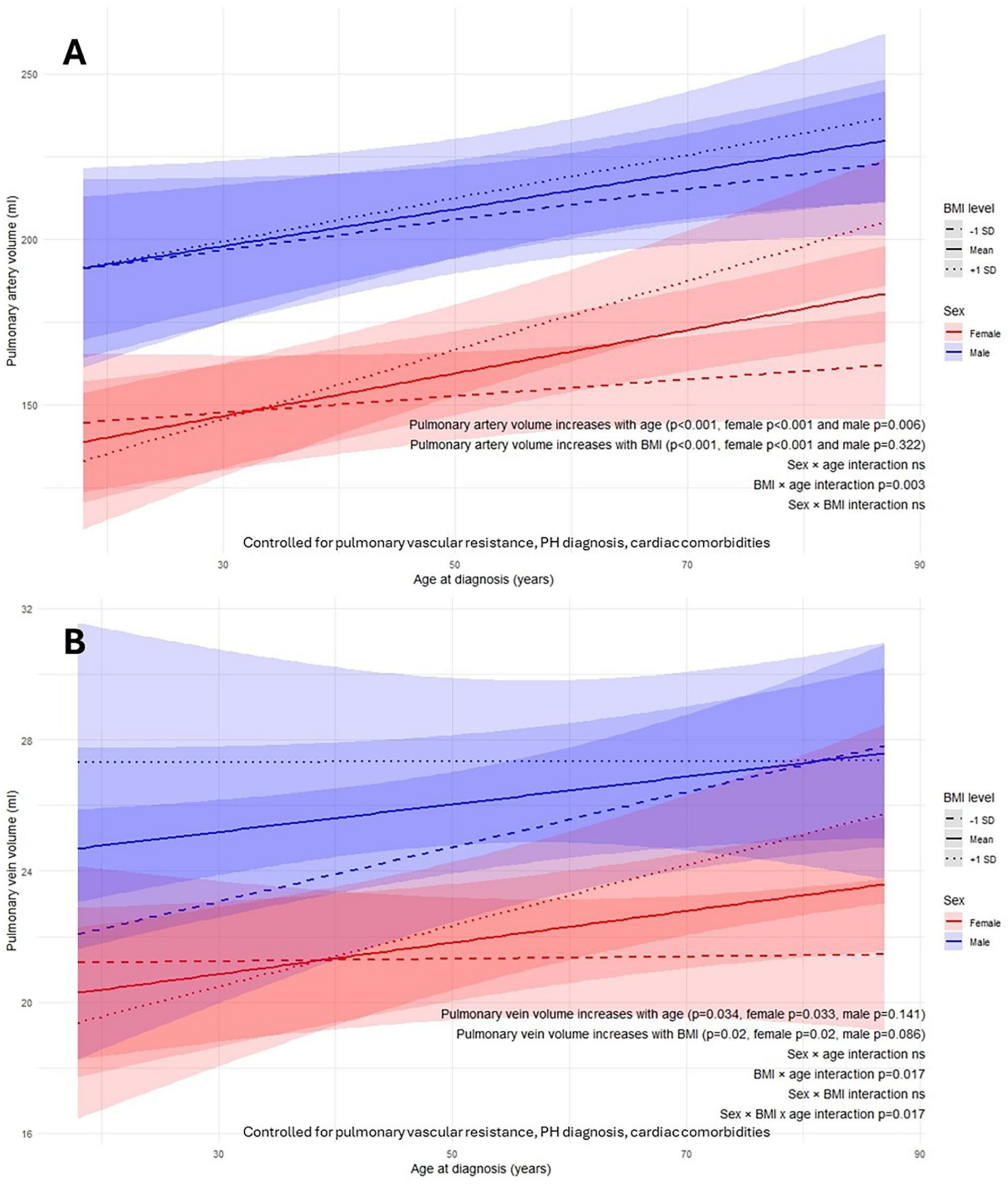
Body mass index (BMI)-sex-age at diagnosis interactions on AI-quantified pulmonary artery volume **(A)** and pulmonary vein volume **(B)** from computed tomography pulmonary angiography. ns, not significant; p: *p*-value; SD, standard deviation.

Pulmonary artery volume increased with age at diagnosis in both females and males (*p* < 0.001) (Figure 5A). In contrast, BMI demonstrated a significant positive association in females (*p* < 0.001), while the corresponding effect in males was attenuated and not statistically significant (*p* = 0.322). A significant diagnosis age–BMI interaction indicated a stronger BMI-related increase in pulmonary artery volume with older diagnosis age (age-BMI interaction *p* = 0.003).

Pulmonary vein volume increased with BMI (*p* = 0.02) and diagnosis age (*p* = 0.034) in females (Figure 5B). Although males demonstrated similar trends with BMI (*p* = 0.086) and diagnosis age (*p* = 0.141), these did not reach statistical significance. The effect of BMI-associated increase in pulmonary vein volume at older diagnosis ages was significantly greater in females than in males (sex-BMI-age interaction *p* = 0.017).

### 3.5 BSA-sex-diagnosis age interaction

**Figure 6:**
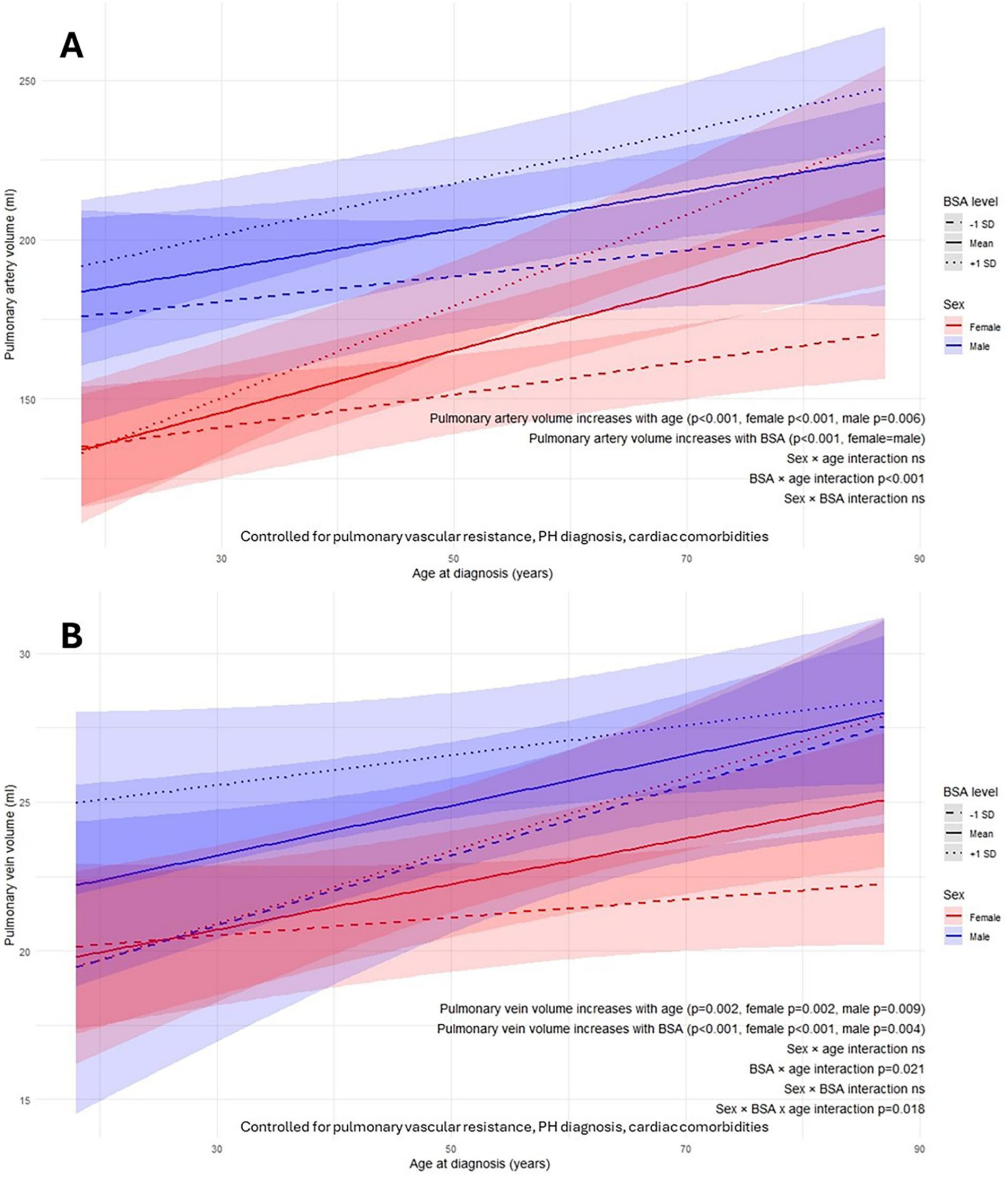
Body surface area (BSA)-sex-age at diagnosis interactions on AI-quantified pulmonary artery volume **(A)** and pulmonary vein volume **(B)** from computed tomography pulmonary angiography. ns, not significant; p, *p*-value; SD, standard deviation.

Pulmonary artery volume increased overall with BSA and age at diagnosis (both *p* < 0.001) in both sexes (Figure 6A). The association between BSA and pulmonary artery volume strengthened with older diagnosis age (age–BSA interaction *p* < 0.001).

Pulmonary vein volume increased overall with BSA (*p* < 0.001) and with age at diagnosis (*p* = 0.002) in both sexes (Figure 6B). The BSA-related increase in pulmonary vein volume at older diagnosis ages was significantly greater in females than males (sex–BSA–age interaction *p* = 0.018).

### 3.6 Anthropometric-sex-age interaction on pulmonary capillary wedge pressure and left heart chamber sizes

In contrast to pulmonary vein volumes on CTPA, similar sex-related interaction patterns were not observed for pulmonary capillary wedge pressure (PCWP) measured at RHC or for left heart chamber dimensions assessed by echocardiography. Specifically, there were no statistically significant three-way interactions between sex, age at diagnosis, and higher weight or BMI for PCWP, left atrial diameter, left atrial area, or left ventricular diameter.

### 3.7 Pulmonary blood volumes normalization

**Figure 7:**
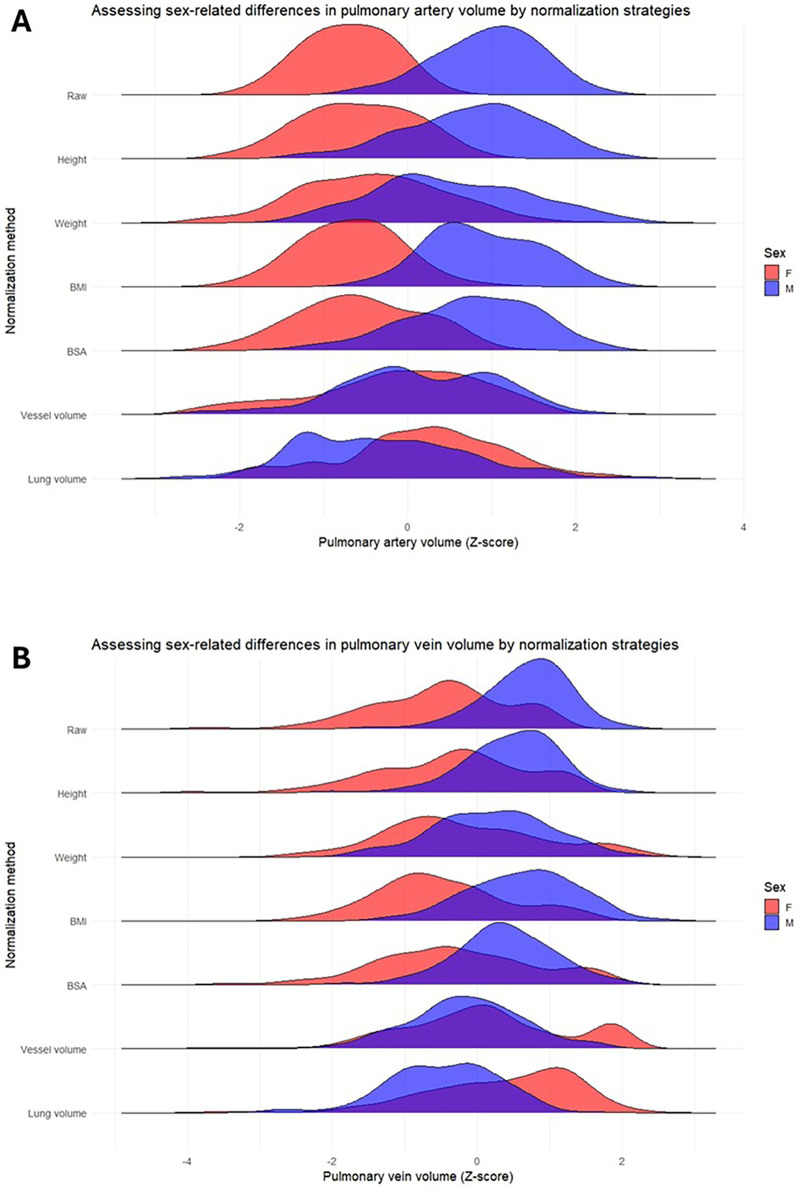
Pulmonary artery **(A)** and vein **(B)** volumes normalization strategies to assess sex-related pulmonary vascular volume differences. BMI, body mass index; BSA, body surface area; F, female; M, male.

Normalization to height, weight, and BSA significantly reduced sex-related differences in both pulmonary artery and vein volumes (all *p* < 0.001) (Figures 7A,B). For pulmonary artery volume, reductions were 0.27 SD units (95% CI: 0.22–0.32) with height, 0.44 SD units (95% CI: 0.28–0.61) with weight, and 0.29 SD units (95% CI: 0.19–0.39) with BSA. Corresponding reductions for pulmonary vein volume were 0.26 SD units (95% CI: 0.22–0.31), 0.38 SD units (95% CI: 0.24–0.52), and 0.30 SD units (95% CI: 0.21–0.38), respectively. In contrast, normalization to BMI did not meaningfully attenuate sex-related differences for either pulmonary arterial (−0.04 SD units, 95% CI: −0.18 to 0.11; *p* = 0.695) or venous volumes (−0.08 SD units, 95% CI: −0.21 to 0.04; *p* = 0.905).

Normalization to total pulmonary vessel volume resulted in near-complete correction of sex-related differences for pulmonary arterial volumes (1.00 SD units, 95% CI: 0.82–1.19) and substantial attenuation for venous volumes (0.80 SD units, 95% CI: 0.66–0.94). In contrast, lung volume normalization consistently led to over-correction of sex-related differences for both pulmonary artery (1.34 SD units, 95% CI: 1.12–1.57) and vein volumes (1.06 SD units, 95% CI: 0.87–1.25).

### 3.8 Pulmonary blood volumes as predictors for PVR and CO

**Figure 8:**
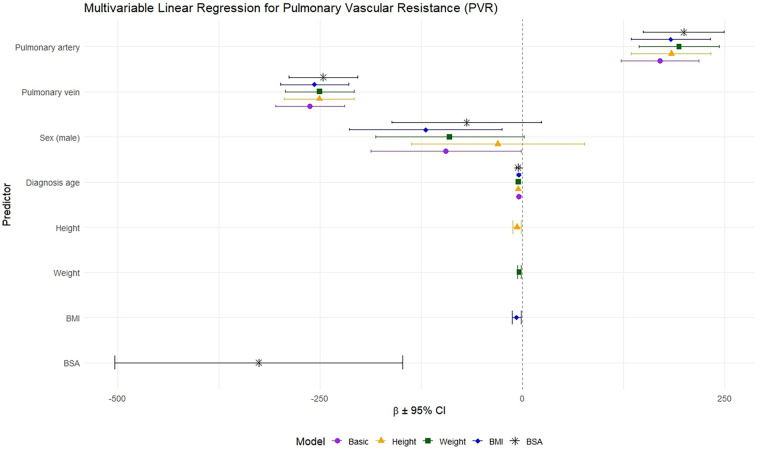
Multivariable linear regression of pulmonary vascular resistance (PVR) on AI-derived pulmonary artery and vein volumes, and anthropometrics. Estimates (β) and 95% confidence intervals (CI) from sequential models are shown: Basic model (pulmonary artery volume, pulmonary vein volume, sex and, diagnosis age), and additional models with height, weight, body mass index (BMI), or body surface area (BSA).

Pulmonary artery and vein volumes were independently associated with PVR, after adjustment for diagnosis age and sex. Addition of anthropometrics significantly improved model fit: height (*F* = 5.44, *p* = 0.02); weight (*F* = 10.49, *p* = 0.001); BMI (*F* = 5.92, *p* = 0.015); and BSA (*F* = 12.85, *p* < 0.001) (Figure 8 and Table 4).

**Table 4:** Multivariable linear regression of pulmonary vascular resistance (PVR) on pulmonary artery and vein volumes, anthropometrics, sex, and diagnosis age. “Basic” model includes pulmonary artery and vein volumes, sex, and diagnosis age; subsequent models additionally include height, weight, BMI, or BSA.

	Basic model		Height model		Weight model		BMI model		BSA model	
Predictor	(β ± SE)	*p*	(β ± SE)	*p*	(β ± SE)	*p*	(β ± SE)	*p*	(β ± SE)	*p*
Pulmonary artery	170.4 ± 24.5	<0.001	184.0 ± 25.0	<0.001	193.8 ± 25.2	<0.001	183.5 ± 24.9	<0.001	199.4 ± 25.4	<0.001
Pulmonary vein	−262.1 ± 21.7	<0.001	−250.8 ± 22.1	<0.001	−249.9 ± 21.7	<0.001	−256.5 ± 21.7	<0.001	−245.8 ± 21.8	<0.001
Sex (male)	−94.5 ± 47.4	0.047	−30.1 ± 54.6	0.582	−89.6 ± 46.8	0.056	−119.1 ± 48.1	0.014	−68.7 ± 47.2	0.146
Diagnosis age	−4.13 ± 1.24	0.001	−5.08 ± 1.30	<0.001	−4.90 ± 1.25	<0.001	−4.28 ± 1.23	<0.001	−5.20 ± 1.26	<0.001
Height	–	–	−6.49 ± 2.78	0.020	–	–	–	–	–	–
Weight	–	–	–	–	−3.59 ± 1.11	0.001	–	–	–	–
BMI	–	–	–	–	–	–	−7.08 ± 2.91	0.015	–	–
BSA	–	–	–	–	–	–	–	–	−325.4 ± 90.7	0.0004

Model performance	Basic Model		Height Model		Weight Model		BMI Model		BSA Model	
*R* ^2^	0.34		0.35		0.36		0.35		0.37	
Compared to basic model, F (*p*-value)	–		5.44 (*p* = 0.02)		10.49 (*p* = 0.001)		5.92 (*p* = 0.015)		12.86 (*p* < 0.001)	

BMI, body mass index; BSA, body surface area.

**Figure 9:**
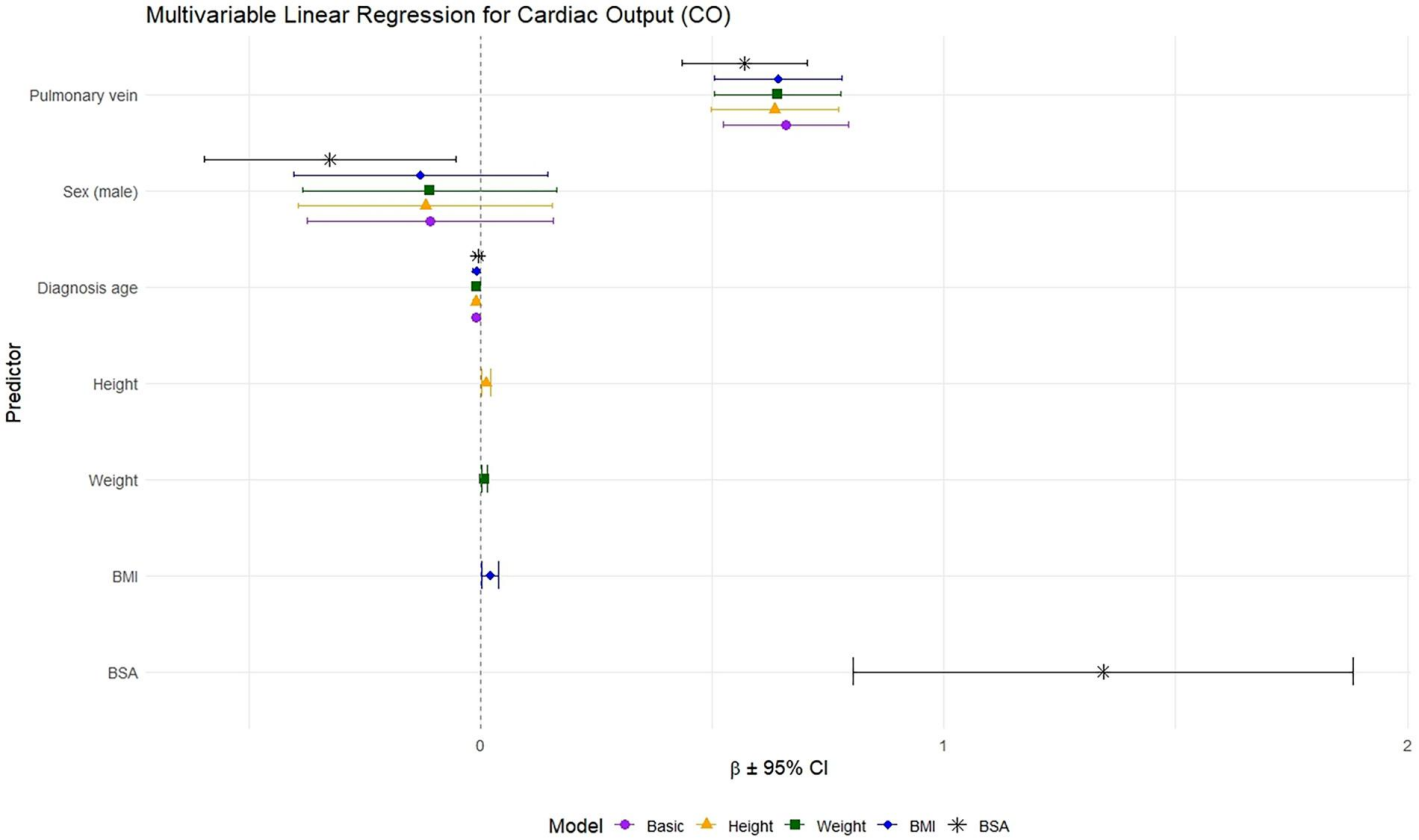
Multivariable linear regression of cardiac output (CO) on AI-derived pulmonary vein volumes and anthropometrics. Estimates (β) and 95% confidence intervals (CI) from sequential models are shown: Basic model (pulmonary vein, sex, and diagnosis age), and additional models with height, weight, body mass index (BMI), or body surface area (BSA).

Pulmonary vein volume was independently associated with CO, adjusted for diagnosis age and sex. Addition of anthropometrics improved model fit: weight (*F* = 23.18, *p* < 0.001); BMI (*F* = 17.61, *p* < 0.001); and BSA (*F* = 23.79, *p* < 0.001) (Figure 9 and Table 5).

**Table 5:** Multivariable linear regression of cardiac output (CO) on pulmonary vein volumes, anthropometrics, sex, and diagnosis age. “Basic” model includes pulmonary vein volume, sex, and diagnosis age; subsequent models additionally include height, weight, BMI, or BSA.

Predictor	Basic model		Height model		Weight model		BMI model		BSA model	
	(β ± SE)	*p*	(β ± SE)	*p*	(β ± SE)	*p*	(β ± SE)	*p*	(β ± SE)	*p*
Pulmonary vein	0.66 ± 0.07	<0.001	0.63 ± 0.07	<0.001	0.58 ± 0.07	<0.001	0.62 ± 0.07	<0.001	0.57 ± 0.07	<0.001
Sex (male)	−0.11 ± 0.14	0.420	−0.27 ± 0.17	0.109	−0.23 ± 0.13	0.087	−0.04 ± 0.13	0.757	−0.33 ± 0.14	0.019
Diagnosis age	−0.01 ± 0.04	0.018	−0.007 ± 0.004	0.062	−0.007 ± 0.004	0.071	−0.01 ± 0.004	0.016	−0.006 ± 0.004	0.109
Height	–	–	−0.01 ± 0.01	0.116	–	–	–	–	–	–
Weight	–	–	–	–	0.016 ± 0.003	<0.001	–	–	–	–
BMI	–	–	–	–	–	–	0.04 ± 0.01	<0.001	–	–
BSA	–	–	–	–	–	–	–	–	1.34 ± 0.28	<0.001

Model performance	Basic Model		Height Model		Weight Model		BMI Model		BSA Model	
R^2^	0.21		0.21		0.25		0.24		0.26	
Compared to basic model, F (*p*-value)	–		2.49 (*p* = 0.116)		23.18 (*p* < 0.001)		17.61 (*p* < 0.001)		23.79 (*p* < 0.001)	

BMI, body mass index; BSA, body surface area.

## 4 Discussion

In this study, we demonstrate the feasibility and clinical relevance of large-scale AI-based post-processing of CTPA for automated pulmonary vascular segmentation and PBV quantification in a well-characterized cohort of patients investigated for PH. The 7th World Symposium on Pulmonary Hypertension highlighted the expanding role of radiological imaging and implementation of AI in non-invasive PH assessment, with a particular emphasis on clinical actionability and interpretability (10). While prior studies have demonstrated an evolving technical feasibility of automated pulmonary vascular segmentation and PBV quantification from CT in PH, they have largely not accounted for interpretation with fundamental physiological determinants such as body size, sex, or diagnosis age (4–9). By leveraging a PH registry with contemporaneous RHC, we provide the first comprehensive evaluation of: 1. how anthropometrics, biological sex, and age at diagnosis influence AI-derived PBV from CTPA; 2. whether normalization strategies can mitigate expected sex-related differences; and 3. how anthropometrics influences prediction of hemodynamics from CTPA-based PBV. Our findings have important implications for the development of physiologically grounded, clinically intuitive CTPA-based PBV-enabled predictive models in PH (10, 11).

### 4.1 Anthropometrics, sex, and diagnosis age as key determinants of PBV

We observed consistent increases in pulmonary artery and vein volumes with height, weight, BMI, and BSA, independent of PVR, different PH diagnostic category, and cardiac comorbidities, supporting the concept that PBV scales with overall body size (16–20). Furthermore, male biological sex was an independent predictor for higher pulmonary artery and vein volumes. This demonstrates the expected physiological effect from a generally larger body size on PBV compared to females. Additionally, older diagnosis age was an independent predictor of higher pulmonary artery and vein volumes. Our finding corroborates with previous studies that show larger main pulmonary artery diameter with age (16–19).

**Figure 10:**
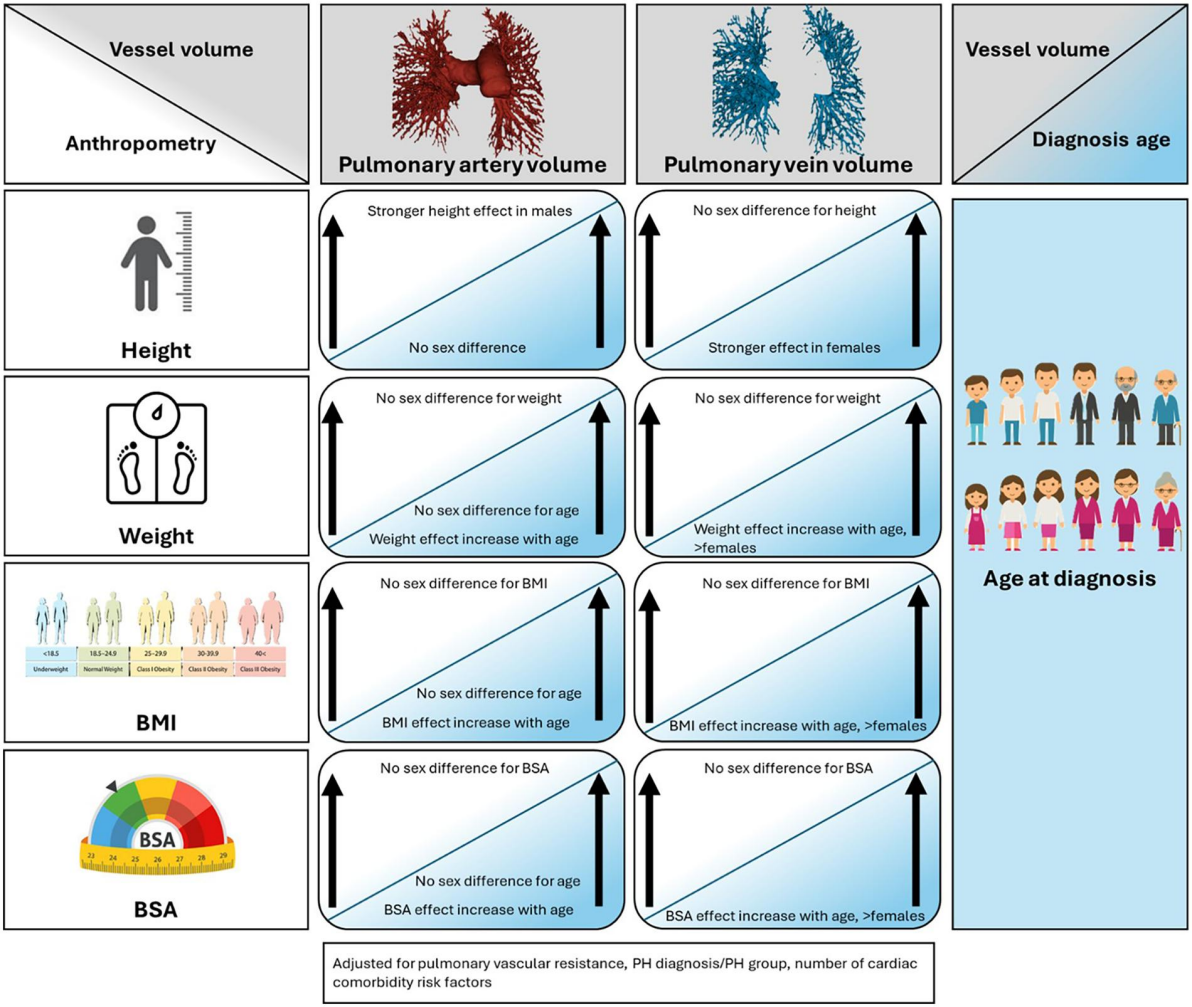
Infographic illustrating the effect of anthropometrics-sex-age at diagnosis interactions on AI-based pulmonary blood volumes quantification from computed tomography pulmonary angiography. BMI, body mass index; BSA, body surface area.

Our novel multivariable models, incorporating anthropometric–sex–diagnosis age interactions, showed that the relationships of anthropometric measures were not uniform across the biological sexes or age at diagnosis but rather have a variable modulating effect on PBV (Figure 10). Notably, height exerted a disproportionately stronger effect on pulmonary artery volume in males, likely reflecting sex-related pulmonary dysanapsis and sexual dimorphism in lung development and morphology (15, 21–23). Sexual dimorphism in lung anatomy is well established, with males exhibiting larger lung volumes and airway dimensions than females, even after adjusting for body size. Pulmonary dysanapsis illustrates that airway size does not scale uniformly with lung volume, and as pulmonary vasculature develops in parallel with airways, these sex-related differences may underlie the stronger height–pulmonary artery volume relationship we observed in males. Additionally, we found that weight-, BMI-, and BSA-associated increases in pulmonary artery volume became more pronounced with older age at diagnosis, a pattern seen in both sexes. The compounding effect on pulmonary artery volume from higher weight and BMI with older diagnosis age and associated comorbidities likely reflected age-related vascular remodeling previously described (17–19).

Pulmonary vein volumes derived from CTPA demonstrated a more complex anthropometric–sex–diagnosis age interactions, suggesting that pulmonary venous remodeling is not merely a scaled analogue of arterial changes but is influenced by sex-specific physiology, body composition, and aging. Although pulmonary vein volume increased with anthropometrics in both sexes, the compounding effect of older age with higher weight, BMI or BSA was more pronounced in females. These sex-related differences are biologically plausible given well-documented differences in cardiovascular aging and ventricular–vascular interactions between females and males (24, 25). With advancing age, females are more likely to develop increased left ventricular stiffness, concentric remodeling, and diastolic dysfunction—all of which alter ventricular filling and venous dynamics, potentially increasing pulmonary venous pressures. Age- and sex-specific differences in vascular stiffness and neurohormonal signaling have also been described and could contribute further to divergent venous remodeling patterns. Moreover, the prevalence of heart failure with preserved ejection fraction (HFpEF)—a condition characterized by impaired ventricular compliance and elevated venous pressures—is higher in older females and is exacerbated by greater adiposity and higher BMI, both of which have been linked to adverse hemodynamic profiles (26). However, these plausible explanations require direct testing using CTPA-derived pulmonary vein volumes. We did not see similar compounding effect of older age with higher weight or BMI in females towards PCWP on RHC or left heart chamber sizes on echocardiography. This may be due to masking of left heart disease on RHC or echocardiography due to impaired left sided preload in our predominantly pre-capillary PH cohort (27). Therefore, further validating these potentially earlier or masked changes seen with pulmonary vein volume on CTPA in older female patients with higher BMI or weight is crucial.

### 4.2 PBV normalization with anthropometrics, total pulmonary vessel volume, and lung volume

An objective of this study was to determine whether normalization of CTPA-derived PBV could reduce sex-related differences, thereby improving comparability at an individual level during PH assessment. We demonstrate that normalization of PBV to height, weight, and BSA substantially attenuated sex-related differences in both pulmonary artery and vein volumes. However, PBV normalization to BMI did not meaningfully reduce sex-related differences. This is physiologically grounded: height, weight, and BSA directly reflect overall body size and cardiopulmonary metrics, therefore more appropriate for scaling PBV (28, 29). Despite anthropometric normalization and adjustment for age at diagnosis, PVR, PH diagnostic category, and cardiac comorbidities, residual differences in PBV persisted between the biological sexes (15). These residual differences likely reflect intrinsic biological variation in pulmonary vascular development, vessel compliance, or cardiac–pulmonary coupling, rather than simply body size (21–25).

PBV normalization to total lung volume resulted in overcorrection of sex-related differences, particularly in males. A recent finding from 11,784 participants with CT scans for pulmonary and cardiovascular diseases showed that females are likely to have greater vessel abundance, in both vessel skeleton length and branch counts, compared to males given the same lung volume (30). This again likely reflects sex-related pulmonary dysanapsis and sexual dimorphism, whereby the scaling relationship between lung size and pulmonary vascular structure differs between sexes (21–23). In this context, lung volume in males may increase disproportionately relative to pulmonary vascular blood volume, such that normalization to lung volume leads to systematic overadjustment in males.

Normalization to total pulmonary vessel volume substantially abolished the difference in PBV between sexes. However, this presumably represents overcorrection due to mathematical coupling between the numerator and denominator as PBV constitutes a component of total pulmonary vessel volume. Such coupling can induce spurious correlations and artificially reduce between-group differences, independent of underlying biology. Consequently, the apparent elimination of sex differences following this normalization should be interpreted with caution, as it may represent a statistical artefact rather than a true absence of biological variation.

### 4.3 Clinical and research implications

The anthropometric–sex–diagnosis age interactions identified in this study underscore that CTPA-derived PBV should not be interpreted as a static structural metric. Rather, PBV represents a dynamic vascular phenotype shaped by body size, biological sex, and temporal factors related to aging and disease evolution. This has important implications for the development and deployment of AI-derived CTPA-based PBV as a biomarker for PH risk prediction and phenotyping. From a clinical perspective, our findings highlight the need to reduce biological and anthropometric contextual blindness in AI-derived PBV measurements to minimize risk of systematic bias, misclassification, and inequitable clinical performance (10–13).

We showed that CTPA-derived PBV reflected hemodynamic burden: larger pulmonary artery volumes, but lower pulmonary vein volumes, were independently associated with higher PVR, suggesting arterial dilation with impaired venous return. Conversely, larger pulmonary vein volumes were associated with higher CO, reflecting preserved or increased pulmonary blood flow. Incorporating anthropometrics, particularly BSA, improved the prediction of invasively measured PVR and CO, supporting the importance anthropometric adjustment when interpreting PBV from CTPA.

PBV normalization strategies may enhance interpretability and promote adoption of explainable AI-derived CTPA metrics in heterogeneous, real-world PH populations (31). Our results suggest that normalization to anthropometrics or explicit adjustment for height, weight, or BSA should be considered when PBV is used in PH prediction models. However, because anthropometric normalization did not fully attenuate sex-related differences in PBV, biological sex should likely be retained as an independent variable rather than assumed to be adequately accounted for by body size alone (21–23). Normalization of PBV to total pulmonary vessel volume yields a compositional metric reflecting the relative distribution of arterial and venous volumes. While this approach may obscure absolute size differences, it may be informative for phenotyping PH etiology, where differential arterial vs. venous remodeling is biologically relevant (9). Our observed interaction effects with diagnosis age support modelling age explicitly—including potential interaction—rather than treating it as a simple linear covariate in predictive frameworks.

### 4.4 Limitations

As this study was a secondary analysis of a prospectively recorded PH database, there were associated limitations. Patients were largely excluded due to inaccessibility of older CTPA images or unavailability of CTPA as part of routine PH assessment. CTPA originating from multiple UK hospitals was a strength allowing generalizability of AI-based vascular segmentation and PBV quantification. However, the lack of standard CTPA acquisition protocol may affect CTPA-derived measurements. Despite adjustment for confounders including PH diagnostic category, PVR, and cardiac comorbidities, residual modulators towards PBV from unmeasured factors such as hormonal influences cannot be excluded. Although we have captured diagnosis age for disease assessment timing, it does not fully capture lifetime exposure to pulmonary vascular remodeling. Furthermore, we were unable to replicate the observed sex–age–anthropometric interactions in pulmonary vein volumes with respect to PCWP on RHC or left heart chamber sizes on echocardiography. Although this discrepancy could reflect the masking and limited sensitivity of RHC and echocardiographic measurements to detect masked left heart disease in this predominantly pre-capillary PH cohort, further studies incorporating provocative testing could clarify this relationship (27). Additionally, we have not investigated if ethnic demography could influence PBV to promote equitable AI-derived metrics. Further research in CTPA-derived PBV is required to comprehend the full effect from demography, disease, and longitudinal changes with aging.

## 5 Conclusions

AI-derived PBV from CTPA are influenced by anthropometrics, sex, and age at diagnosis, with complex interaction effects that differ between pulmonary arteries and veins. Normalization to anthropometrics substantially attenuate, but does not eliminate, sex-related differences in PBV. Furthermore, incorporating anthropometrics into PBV-based models improves the prediction of invasively measured hemodynamics. These findings highlight the importance of accounting for biological and anthropometric determinants, bridging a critical knowledge gap when translating AI-quantified CTPA-derived PBV into clinically intuitive PH prediction and phenotyping models.

## Data Availability

The raw data supporting the conclusions of this article will be made available by the authors, without undue reservation.

## References

[1] Kovacs G Bartolome S Denton CP Gatzoulis MA Gu S Khanna D Definition, classification and diagnosis of pulmonary hypertension. Eur Respir J. (2024) 64:2401324. 10.1183/13993003.01324-2024 39209475 PMC11533989

[2] Ghani H Weir-McCall JR Ruggiero A Pepke-Zaba J. Imaging in chronic thromboembolic pulmonary disease: current practice and advances. Int J Cardiol Congenit Heart Dis. (2024) 17:100536. 10.1016/j.ijcchd.2024.100536 39711768 PMC11657945

[3] Remy-Jardin M Ryerson CJ Schiebler ML Leung ANC Wild JM Hoeper MM Imaging of pulmonary hypertension in adults: a position paper from the fleischner society. Radiology. (2021) 298(3):531–49. 10.1148/radiol.2020203108 33399507

[4] Ghani H Thillai M Jenkins D Bussell E Ruggiero A Walsh S Pulmonary blood volumes on CT predict residual pulmonary hypertension post-pulmonary endarterectomy. Am J Respir Cell Mol Biol. (2025). 10.1165/rcmb.2025-0121OC 42085489

[5] Rahaghi FN Nardelli P Harder E Singh I Sánchez-Ferrero GV Ross JC Quantification of arterial and venous morphologic markers in pulmonary arterial hypertension using CT imaging. Chest. (2021) 160(6):2220–31. 10.1016/j.chest.2021.06.069 34270966 PMC8692106

[6] Melzig C Wörz S Egenlauf B Partovi S Rohr K Grünig E Combined automated 3D volumetry by pulmonary CT angiography and echocardiography for detection of pulmonary hypertension. Eur Radiol. (2019) 29(11):6059–68. 10.1007/s00330-019-06188-7 30963276

[7] Rahaghi FN Ross JC Agarwal M González G Come CE Diaz AA Pulmonary vascular morphology as an imaging biomarker in chronic thromboembolic pulmonary hypertension. Pulm Circ. (2016) 6(1):70–81. 10.1086/685081 27162616 PMC4860553

[8] Pienn M Gertz RJ Gerhardt F Kröger JR Zaytoun H Reimer RP CT-derived lung vessel morphology correlates with prognostic markers in pre-capillary pulmonary hypertension. J Heart Lung Transplant. (2023) 43:54–65. 10.1016/j.healun.2023.08.013 37619642

[9] Synn AJ Harder EM Nardelli P Ross JC Maron BA Leopold JA Automated CT-based quantification of pulmonary veins shows greater central venous dilation in group 2 pulmonary hypertension compared with group 1 pulmonary arterial hypertension and control subjects. CHEST Pulmonary. (2023) 1(3):100020. 10.1016/j.chpulm.2023.100020 38144213 PMC10745213

[10] Rajagopal S Bogaard HJ Elbaz MSM Freed BH Remy-Jardin M van Beek EJR Emerging multimodality imaging techniques for the pulmonary circulation. Eur Respir J. (2024) 64:2401128. 10.1183/13993003.01128-2024 39209480 PMC11525339

[11] Ghassemi M Oakden-Rayner L Beam AL. The false hope of current approaches to explainable artificial intelligence in health care. Lancet Digit Health. (2021) 3(11):e745–50. 10.1016/S2589-7500(21)00208-9 34711379

[12] Behzad S Tabatabaei SMH Lu MY Eibschutz LS Gholamrezanezhad A. Pitfalls in interpretive applications of artificial intelligence in radiology. Am J Roentgenol. (2024) 223(4):e2431493. 10.2214/AJR.24.31493 39046137

[13] Yi PH Bachina P Bharti B Garin SP Kanhere A Kulkarni P Pitfalls and best practices in evaluation of AI algorithmic biases in radiology. Radiology. (2025) 315(2). 10.1148/radiol.241674 40392092 PMC12127964

[14] Ventetuolo CE Praestgaard A Palevsky HI Klinger JR Halpern SD Kawut SM. Sex and haemodynamics in pulmonary arterial hypertension. Eur Respir J. (2014) 43(2):523–30. 10.1183/09031936.00027613 23949961 PMC4338984

[15] Wright SP Kirby M Singh GV Tan WC Bourbeau J Eves ND. Sex-related differences in pulmonary vascular volume distribution. Pulm Circ. (2024) 14(3). 10.1002/pul2.12436 PMC11391118 39268397

[16] Truong QA Massaro JM Rogers IS Mahabadi AA Kriegel MF Fox CS Reference values for normal pulmonary artery dimensions by noncontrast cardiac computed tomography. Circ Cardiovasc Imaging. (2012) 5(1):147–54. 10.1161/CIRCIMAGING.111.968610 22178898 PMC3275437

[17] Paul TK Alamin AE Subedi P Zhang M Diab MM Alamian A Association between cardiovascular risk factors and the diameter of the main pulmonary artery in asymptomatic population in the Appalachian region. J Thorac Dis. (2019) 11(8):3435–42. 10.21037/jtd.2019.08.09 31559048 PMC6753455

[18] Burman ED Keegan J Kilner PJ. Pulmonary artery diameters, cross sectional areas and area changes measured by cine cardiovascular magnetic resonance in healthy volunteers. J Cardiovasc Magn Reson. (2016) 18(1):12. 10.1186/s12968-016-0230-9 26940894 PMC4778312

[19] Lee SH Kim YJ Lee HJ Kim HY Kang YA Park MS Comparison of CT-determined pulmonary artery diameter, aortic diameter, and their ratio in healthy and diverse clinical conditions. PLoS One. (2015) 10(5):e0126646. 10.1371/journal.pone.0126646 25955036 PMC4425684

[20] Berger T Siepe M Simon B Beyersdorf F Chen Z Kondov S Pulmonary artery diameter: means and normal limits—assessment by computed tomography angiography. Interact Cardiovasc Thorac Surg. (2022) 34(4):637–44. 10.1093/icvts/ivab308 34791257 PMC9026207

[21] Sheel AW Dominelli PB Molgat-Seon Y. Revisiting dysanapsis: sex-based differences in airways and the mechanics of breathing during exercise. Exp Physiol. (2016) 101(2):213–8. 10.1113/EP085366 26440369

[22] Torres-Tamayo N García-Martínez D Lois Zlolniski S Torres-Sánchez I García-Río F Bastir M. 3D Analysis of sexual dimorphism in size, shape and breathing kinematics of human lungs. J Anat. (2018) 232(2):227–37. 10.1111/joa.12743 29148039 PMC5770305

[23] Bellemare F Jeanneret A Couture J. Sex differences in thoracic dimensions and configuration. Am J Respir Crit Care Med. (2003) 168(3):305–12. 10.1164/rccm.200208-876OC 12773331

[24] Oneglia A Nelson MD Merz CNB. Sex differences in cardiovascular aging and heart failure. Curr Heart Fail Rep. (2020) 17(6):409–23. 10.1007/s11897-020-00487-7 32984923 PMC7724574

[25] Kaur G Lau E. Sex differences in heart failure with preserved ejection fraction: from traditional risk factors to sex-specific risk factors. Womens Health. (2022) 18:17455057221140208. 10.1177/17455057221140209 PMC9720805 36448661

[26] Sorimachi H Omote K Omar M Popovic D Verbrugge FH Reddy YNV Sex and central obesity in heart failure with preserved ejection fraction. Eur J Heart Fail. (2022) 24(8):1359–70. 10.1002/ejhf.2563 35599453 PMC12276836

[27] Maron BA Bortman G De Marco T Huston JH Lang IM Rosenkranz SH Pulmonary hypertension associated with left heart disease. Eur Respir J. (2024) 64:2401344. 10.1183/13993003.01344-2024 39209478 PMC11525340

[28] Fung ASY Soundappan D Loewenstein DE Playford D Strange G Kozor R Prognostic association supports indexing size measures in echocardiography by body surface area. Sci Rep. (2023) 13(1):19390. 10.1038/s41598-023-46183-z 37938592 PMC10632399

[29] Madronio CM Pathan S Low GKK Nundlall N Williams K Badorrek S The effect of body size and obesity on cardiovascular haemodynamics and myocardial mechanics. Heart Lung Circ. (2025) 34(10):1109–18. 10.1016/j.hlc.2025.08.014 40945959

[30] Chu Y Luo G Zhou L Cao S Ma G Meng X Deep learning-driven pulmonary artery and vein segmentation reveals demography-associated vasculature anatomical differences. Nat Commun. (2025) 16(1):2262. 10.1038/s41467-025-56505-6 40050617 PMC11885638

[31] Ghani H Pepke-Zaba J. Navigating between management of pulmonary arterial hypertension and cardiometabolic and pulmonary comorbidities. J Heart Lung Transplant. (2025) 44(2):147–9. 10.1016/j.healun.2024.09.016 39395468

