# Supplementary material for: Anthropometry, sex, and age at diagnosis affect pulmonary blood volume quantification from computed tomography pulmonary angiography in pulmonary hypertension assessment
Source: Front Cardiovasc Med. 2026 May 11;13:1815977. doi: 10.3389/fcvm.2026.1815977 (PMC13198986; doi:10.3389/fcvm.2026.1815977)
Supplement: Supplementary file 1 [file Table1.docx]

Anthropometry, sex, and age at diagnosis affect pulmonary blood volume quantification from computed tomography pulmonary angiography in pulmonary hypertension assessment

Authors: Hakim Ghani^1,2^, Muhunthan Thillai^3,4^, Simon Walsh^4,5^, Elliott Bussell^5^, Martin Graves^2^, Joanna Pepke-Zaba^1,2^

Affiliations:

^1^National Pulmonary Hypertension Centre, Pulmonary Vascular Disease Unit, Royal Papworth Hospital, Cambridge, UK

^2^University of Cambridge, UK

^3^ Interstitial Lung Diseases Unit, Royal Papworth Hospital, Cambridge, UK

^4^Qureight Ltd, Cambridge, UK

^5^National Heart and Lung Institute, Imperial College, London, UK

Supplement

Supplement Figures and Tables

Table S1. Strongest correlations between CTPA metrics and anthropometrics

| CTPA metrics | Anthropometrics | r_s_ | Adjusted p-value |
| --- | --- | --- | --- |
| Central pulmonary vessel volume | BSA | 0.4 | <0.001 |
| Artery to vein volume ratio | BMI | 0.4 | <0.001 |
| Total artery volume | BSA | 0.39 | <0.001 |
| Central pulmonary vessel volume | Weight | 0.39 | <0.001 |
| Central pulmonary artery volume | BSA | 0.38 | <0.001 |
| Central pulmonary artery volume | Weight | 0.37 | <0.001 |
| Total artery volume | Weight | 0.37 | <0.001 |
| Artery to vein volume ratio | Weight | 0.37 | <0.001 |
| Total artery volume per lung volume | BMI | 0.36 | <0.001 |
| Artery to vein volume ratio | BSA | 0.35 | <0.001 |
| Lung volume | Height | 0.34 | <0.001 |
| Total vessel volume | Height | 0.33 | <0.001 |
| Central pulmonary vessel volume | BMI | 0.32 | <0.001 |
| Total vessel volume per lung volume | BMI | 0.31 | <0.001 |
| Central pulmonary artery volume | BMI | 0.31 | <0.001 |
| Total artery volume | Height | 0.31 | <0.001 |
| Total vessel volume | BSA | 0.3 | <0.001 |
| Intrapulmonary artery volume | Height | 0.28 | <0.001 |
| Main pulmonary artery diameter | BSA | 0.28 | <0.001 |
| Total artery volume | BMI | 0.28 | <0.001 |
| Total artery volume per lung volume | Weight | 0.27 | <0.001 |
| Intrapulmonary vessel volume | Height | 0.27 | <0.001 |
| Total vessel volume | Weight | 0.27 | <0.001 |
| Total vein volume | Height | 0.27 | <0.001 |
| Correlations adjusted for biological sex, age at diagnosis, pulmonary vascular resistance, and pulmonary hypertension (PH) diagnostic category (group 1, 2, 3 or 4 PH, or without PH).  Adjusted p-value for multiple testing using the FDR method  BMI: body mass index; BSA: body surface area | | | |
