# Supplementary material for: Anthropometry, sex, and age at diagnosis affect pulmonary blood volume quantification from computed tomography pulmonary angiography in pulmonary hypertension assessment
Source: Front Cardiovasc Med. 2026 May 11;13:1815977. doi: 10.3389/fcvm.2026.1815977 (PMC13198986; doi:10.3389/fcvm.2026.1815977)
Supplement: Supplementary file 2 [file Supplementaryfile1.docx]

Anthropometry, sex, and age at diagnosis affect pulmonary blood volume quantification from computed tomography pulmonary angiography in pulmonary hypertension assessment

Authors: Hakim Ghani^1,2^, Muhunthan Thillai^3,4^, Simon Walsh^4,5^, Elliott Bussell^5^, Martin Graves^2^, Joanna Pepke-Zaba^1,2^

Affiliations:

^1^National Pulmonary Hypertension Centre, Pulmonary Vascular Disease Unit, Royal Papworth Hospital, Cambridge, UK

^2^University of Cambridge, UK

^3^ Interstitial Lung Diseases Unit, Royal Papworth Hospital, Cambridge, UK

^4^Qureight Ltd, Cambridge, UK

^5^National Heart and Lung Institute, Imperial College, London, UK

Supplement

Extended methods

*Patient cohort and clinical data*

All patients who were investigated for pulmonary hypertension (PH) with right heart catheterization (RHC) at Royal Papworth Hospital were eligible to be recruited for this study. The CAPHTURE (Cambridge PH Registry) database consists of prospectively recorded routine investigations, management, and outcomes of PH patients investigated and discussed at the multidisciplinary team meeting based at Royal Papworth Hospital. Consecutive patients were recruited to this study by screening the available CAPHTURE database for patients who underwent PH assessment between 2001-2024. Due to availability of different PH groups in the database, patients who were assessed for PH and found to have pulmonary arterial hypertension (PAH), group 2 PH, group 3 PH, chronic thromboembolic PH (CTEPH), and without PH were recruited between 2001-2003, 2020-2022, 2012-2024, 2020-2022, and 2015-2024 respectively.

The inclusion criteria for patients recruited into this study were:

1. Patients 18 years of age or older at time of investigation
2. Investigated for PH with RHC and with hemodynamic data readily available
3. Computed tomography pulmonary angiography (CTPA) within four months of RHC, CTPA slice <2 mm and good quality (without significant motion artifact, poor contrast enhancement or limited view of the pulmonary vasculature)
4. CTPA and RHC before initiation of PH management

The exclusion criteria were:

1. Consent withdrawn for anonymized data usage through the National Health Service (NHS) National Data Opt-outs Service
2. Referral originated from outside the UK

Patients were largely excluded due to inaccessibility of older CTPA images. CTPA images were independently assessed by both a Radiologist and a clinical research fellow for quality. Quality of CTPA images were assessed for: 1. minimal motion artifact; 2. good contrast enhancement and 3. complete view of the pulmonary vasculature). If CTPA images were assessed to be of good quality for all three of these inclusion parameters, patients were included in this study. Pre-specified criteria for good quality CTPA within a limited time frame to RHC hemodynamic measurements were required to ensure clinically interpretable pulmonary blood volume (PBV) data in relation to PH assessment.

Clinical information that was extracted from the CAPHTURE database were RHC hemodynamic measurements, six-minute walk distance, World Health Organization functional class, NTproBNP, lung function test, patient demographic, and anthropometrics.

*Study objectives*

In patients investigated for PH, this study aimed to use AI-based CTPA-derived PBV quantification to:

1. Characterize the independent and interacting effects of anthropometrics, biological sex, and age of PH diagnosis on pulmonary artery and vein volumes.
2. Evaluate whether sex-related differences in PBV are attenuated by normalization to anthropometrics, total pulmonary vessel volume, or lung volume.
3. Determine whether anthropometric factors influence the associations between PBV and invasively measured PVR and CO

*Automated pulmonary vascular segmentation from CTPA and pulmonary blood volumes measurements*

Automated pulmonary vasculature segmentation from lung parenchyma with differentiation of arteries and veins was performed by a three-dimensional convolutional neural network-based model (Vascul8^TM^, Qureight Ltd.) – an experimental extension of the vessel model previously described and published for contrast-enhanced scans [1, 2]. This new model was trained on segmentations of pulmonary vessels separating arteries and veins, performed by board-certified thoracic radiologists. These segmentations were carried out by manually segmenting the central vessels, then using a region growing algorithm to grow those central vessels into the thresholded vesselness output (described in Ghani et al and Thillai et al, 2024) [1, 2]. Finally, extensive review and further manual corrections were applied by the radiologists. Further details of Vascul8^TM^ were previously published by Thillai et al, 2024 [2].

Automated detection of pulmonary blood vessels was up to the visible limitation of CTPA images (about 2 mm). On manual inspection, pulmonary arterial and venous detection were approximately similar. Performance of AI-based CTPA segmentation and automated PBV quantification in a PH population were previously performed and reported by Ghani et al, 2025 [1].

Pulmonary arteries and veins were additionally automatically compartmentalized by boundaries of the lung mask into those labelled as “central” (up to proximal lobar vessels) and “intrapulmonary” (predominantly segmental and subsegmental), and blood volumes evaluated separately. A total of 12 AI-based CTPA-derived metrics were measured:

1. Total vessel volume
2. Total artery volume
3. Total vein volume
4. Intrapulmonary vessel volume
5. Intrapulmonary artery volume
6. Intrapulmonary vein volume
7. Central pulmonary vessel volume
8. Central pulmonary artery volume
9. Central pulmonary vein volume
10. Intrapulmonary to central pulmonary artery volume ratio
11. Artery to vein volume ratio
12. Lung volume

*Statistical analysis*

Statistical analyses were performed using R (version 4.3.3). Continuous variables were compared between groups using the non-parametric Wilcoxon rank-sum test, and categorical variables were compared using Fisher’s exact test. Associations between anthropometric measures and CTPA-derived pulmonary vascular variables were assessed using Spearman rank correlation coefficients (rₛ). Partial correlations were estimated with adjustment for biological sex, age at diagnosis, pulmonary vascular resistance (PVR), and PH diagnostic category (pulmonary arterial hypertension, group 2 PH, group 3 PH, CTEPH, or without PH).

To account for multiple testing, p-values were adjusted using the false discovery rate (FDR) method, with an FDR-adjusted p-value <0.05 considered statistically significant.

PBV were analyzed using multivariable linear regression. All models included biological sex, age at diagnosis, PVR, PH diagnostic category, and number of cardiac comorbidities as covariates. Anthropometric variables (height, weight, body surface area [BSA], and body mass index [BMI]) were initially examined separately as independent predictors. Interactions between anthropometric variables, sex, and age at diagnosis were also assessed to evaluate potential effect modification. This multivariable linear regression is represented as: Pulmonary blood volume (artery or vein) = β_0_ + β_1_ anthropometry + β_2_ sex + β_3_ diagnosis age + β_4_ (anthropometry​⋅sex) + β_5_ (anthropometry​⋅diagnosis age) + β_6_ (sex​⋅diagnosis age) + β_7_ (anthropometry​⋅sex​⋅diagnosis age) + β_8_ PVR + β_9_ PH diagnostic category + β_10_ Number of cardiac comorbidities ​+ε

To quantify the extent to which normalization strategies reduced sex-related differences in pulmonary artery and vein volumes, raw PBV and PBV normalized to height, weight, BSA, BMI, lung volume, or total pulmonary vessel volume were modelled separately using identical covariate structures. Sex effects from each model were standardized by dividing the estimated sex regression coefficient by the standard deviation of the corresponding outcome, yielding effect sizes in standard deviation (z-score) units.

Differences between standardized sex effects from raw and normalized models were estimated using non-parametric bootstrap resampling (5,000 iterations). For each bootstrap sample, models were refitted and the difference in standardized sex coefficients was recalculated. Statistical inference was based on percentile-based 95% confidence intervals and empirical two-sided p-values derived from the bootstrap distributions. The proportion of the sex-related difference explained by normalization was calculated as the relative reduction in standardized sex effect compared with the raw PBV model.

Pulmonary vascular resistance was modelled using multivariable linear regression with standardized pulmonary arterial and venous volumes (z-scores) as primary predictors. All models were adjusted for age (centered) and sex. To assess the influence of anthropometrics, separate models additionally included, height, weight, BMI, or BSA. Model fit was compared using nested likelihood ratio tests (ANOVA). Similar assessment was performed where cardiac output was modelled using multivariable linear regression with standardized pulmonary venous volumes (z-scores) as primary predictors. The pulmonary vascular resistance model is represented as: PVR = β_0_ + β_1_ pulmonary artery blood volume + β_2_ pulmonary vein blood volume + β_3_ sex + β_4_ diagnosis age + β_4_ anthropometry + ε. The cardiac output model is represented as: CO = β_0_ + β_1_ pulmonary vein blood volume + β_2_ sex+ β_3_ diagnosis age + β_4_ anthropometry + ε.

*Reference*

1. Ghani H, Thillai M, Jenkins D, et al. Pulmonary Blood Volumes on CT Predict Residual Pulmonary Hypertension Post-Pulmonary Endarterectomy. Am J Respir Cell Mol Biol 2025.
2. Thillai M, Oldham JM, Ruggiero A, et al. Deep Learning–based Segmentation of Computed Tomography Scans Predicts Disease Progression and Mortality in Idiopathic Pulmonary Fibrosis. Am J Respir Crit Care Med 2024;210:465–472.
